# Laying sequence interacts with incubation temperature to influence rate of embryonic development and hatching synchrony in a precocial bird

**DOI:** 10.1371/journal.pone.0191832

**Published:** 2018-01-26

**Authors:** Gary R. Hepp, Robert A. Kennamer

**Affiliations:** 1 School of Forestry and Wildlife Sciences, Auburn University, Auburn, Alabama, United States of America; 2 Savannah River Ecology Laboratory, Aiken, South Carolina, United States of America; Gaziosmanpasa University, TURKEY

## Abstract

Incubation starts during egg laying for many bird species and causes developmental asynchrony within clutches. Faster development of late-laid eggs can help reduce developmental differences and synchronize hatching, which is important for precocial species whose young must leave the nest soon after hatching. In this study, we examined the effect of egg laying sequence on length of the incubation period in Wood Ducks (***Aix sponsa***). Because incubation temperature strongly influences embryonic development rates, we tested the interactive effects of laying sequence and incubation temperature on the ability of late-laid eggs to accelerate development and synchronize hatching. We also examined the potential cost of faster development on duckling body condition. Fresh eggs were collected and incubated at three biologically relevant temperatures (Low: 34.9°C, Medium: 35.8°C, and High: 37.6°C), and egg laying sequences from 1 to 12 were used. Length of the incubation period declined linearly as laying sequence advanced, but the relationship was strongest at medium temperatures followed by low temperatures and high temperatures. There was little support for including fresh egg mass in models of incubation period. Estimated differences in length of the incubation period between eggs 1 and 12 were 2.7 d, 1.2 d, and 0.7 d at medium, low and high temperatures, respectively. Only at intermediate incubation temperatures did development rates of late-laid eggs increase sufficiently to completely compensate for natural levels of developmental asynchrony that have been reported in Wood Duck clutches at the start of full incubation. Body condition of ducklings was strongly affected by fresh egg mass and incubation temperature but declined only slightly as laying sequence progressed. Our findings show that laying sequence and incubation temperature play important roles in helping to shape embryo development and hatching synchrony in a precocial bird.

## Introduction

Incubation is an important stage of reproduction in birds during which parents seek to balance their needs with those of developing embryos. Evidence from a variety of species suggests that maintaining eggs at an optimal temperature between 35.5°C and 38.5°C during incubation is critically important for proper embryo development [1]. Eggs incubated at low temperatures, for example, develop more slowly and produce neonates of reduced quality and fitness [2–4]. Alternatively, high incubation temperature shortens the incubation period, but accelerated embryonic development can also be costly to young birds by increasing oxidative damage [5–7].

Incubation starts during egg laying for many bird species and is called partial incubation [8,9]. In precocial species, like prairie-nesting ducks (*Anas* spp.), females increase nest attendance as egg-laying progresses, and egg temperatures can reach levels for effective incubation with laying of the second egg [10]. The potential adaptive value of partial incubation may vary among species [9]. For example, viability of early-laid eggs begins to decline in as little as 3 days, and partial incubation may play an important role in maintaining egg viability [11–13]. Partial incubation may also shorten the incubation period, thereby reducing predation risk and resulting in an earlier hatch date [10,14]. In altricial species, partial incubation produces a size hierarchy within broods that may aid brood reduction during food shortages and improve reproductive success [15], but see [16].

In precocial species, partial incubation can cause developmental asynchrony within clutches of 2 to 3 days at the end of egg-laying [17–18]. However, asynchronous hatching is not desirable because precocial young often must leave the nest within 24 hr of hatching. Eggs of some precocial birds laid later in the sequence have been found to develop faster than early-laid eggs which helps reduce developmental differences and synchronize hatching [19–21]. However, there may be an upper limit to how fast embryos can grow to synchronize hatching [17,22]. Accelerated development of eggs laid later in the sequence may be facilitated by several intrinsic factors including: 1) higher levels of yolk androgens [23,24], 2) differences in egg size and composition [25,26], and 3) increased embryonic metabolic rates [20,21]. Potential costs to neonates of faster development, like the harmful effects of high androgen levels on immune function, may be offset by advantages accruing from partial incubation [27].

Wood Ducks (*Aix sponsa*) nest in cavities, lay 1 egg day^-1^, clutch sizes of nonparasitized nests average 10–12 eggs, and females alone incubate eggs [28]. Partial incubation begins at night 3–4 d before the clutch is complete and full incubation begins resulting in > 2 days of intraclutch developmental asynchrony [14,17,29].

In this study, we examined the effect of egg laying sequence and incubation temperature on length of the incubation period in Wood Ducks. We predicted that eggs laid later in the sequence would have shorter incubation periods than eggs laid earlier thereby helping to reduce developmental asynchrony and synchronize hatching of ducklings. However, we know that rate of embryo development in Wood Ducks varies with incubation temperature [30,31]. Therefore, we tested the interactive effects of laying sequence and incubation temperature on the ability of late-laid eggs to accelerate development and potentially synchronize hatching with early-laid eggs. If there is an upper limit to the development rate of Wood Duck embryos, then we predicted eggs laid later in the sequence and incubated at high temperatures would have less capacity to accelerate development and synchronize hatching with early-laid eggs. We also examined the potential cost of accelerated development on body condition of neonates. If late-laid eggs experience faster development, then intrinsic factors like differences in metabolic rate or allocation of egg nutrients may cause these embryos to hatch in poorer condition than neonates from early-laid eggs.

## Methods

### Study area, field methods and artificial incubation

We conducted the study at the Department of Energy’s Savannah River Site (800 km^2^; SRS) in the upper coastal plain of west-central South Carolina (33.2878°N, 81.723°W). Nest boxes were distributed along the perimeters of Par Pond (*n* = 80; 1120 ha) and L Lake (*n* = 30; 450 ha) and checked every four days during the breeding season (Jan–July) to locate new nests (i.e., eggs were first discovered in the nest). After location of a new nest, the nest was checked daily until egg-laying stopped and full incubation began. We collected eggs from new nests on the day they were discovered and every day subsequent to that until the clutch was completed. Fresh eggs were removed, individually marked with date of collection and nest box identification, and replaced with wooden eggs to prevent females from abandoning nests. If multiple eggs occurred in newly discovered nests, they were randomly assigned a laying sequence number beginning with the first egg. Thereafter, active nests were visited daily to collect eggs and when nests contained >1 egg on daily visits, these eggs were assigned the same laying sequence number. Females normally lay 1 egg day ^-1^, so date of nest initiation was estimated by subtracting the number of eggs in the nest when it was first discovered from the day the nest was checked and adding one. Conspecific brood parasitism is common in Wood Ducks, so if the number of eggs was greater than the number of days between box checks, the day after the previous box check was assumed the nest initiation date. Clutch size was determined at the end of laying.

Fresh eggs were brought back to the lab, weighed (0.01 g), and stored at 20°C which is below physiological zero (24–27°C) when avian embryos begin to develop [1]. Eggs were placed in incubators (Grumbach model BSS 420, Lyon Technologies Inc.) every 4 days, which corresponded with the frequency of new nest box checks. Eggs were incubated at three temperatures (34.9°C, 35.8°C, and 37.6°C; relative humidity = 55–60%) that were within the range of natural incubation temperatures of Wood Ducks [30,32]. Eggs were turned hourly, and incubators were programmed with two 1-hr cool down periods each day to simulate the natural incubation behavior of Wood Ducks [30]. Temperatures declined about 3°C during cool down periods. Data loggers (HOBO® Pro V2, Onset Computer Corp.) were placed in incubators and recorded temperature every 6 minutes. Mean incubation temperatures were calculated each day, and the grand mean temperature over the entire incubation period was calculated for each egg used in our analyses.

We checked and candled eggs regularly to monitor embryo development during incubation. As eggs neared hatching, we checked them twice daily, and all pipped eggs were moved to a single incubator for hatching (37.5°C and 80% humidity). Pipped eggs were placed in individual PVC cylinders (10.6 x 10.6 cm) with mesh tops that enabled us to match eggs with ducklings after hatching. Incubation period was the number of days between when an egg was placed into the incubator until it hatched. Within 24 hr of hatching, ducklings were weighed after allowing them to dry (0.01 g), and tarsus was measured (0.01 mm) with digital calipers. Our protocols for nest box checks and the collection, storage, and incubation of eggs at 4-day intervals also allowed us in another study to examine effects of incubation temperature on subsequent survival and recruitment of ducklings after their placement with foster mothers [31].

### Data analysis

We tested the effect of egg laying sequence on length of incubation period at three incubation temperatures (34.9°C, 35.8°C, and 37.6°C) using mixed linear models [33]. Eggs from individual clutches were incubated at a single temperature. We used models with random intercepts and random slopes to help produce unbiased SEs [34]. We grouped individual nests within temperature treatments and included fresh egg mass as a covariate. Temperature treatment and nests were treated as categorical variables while laying sequence and egg mass were continuous. Laying sequence and egg mass were centered on their grand means. We compared models using Akaike’s Information Criterion corrected for small sample size (AIC_c_) [35]. We ranked models based on their relative differences to the top model (ΔAIC_c_). Akaike weights (*w*_*i*_) are the relative likelihood of the models given the data and model sets. The parameter likelihood is the sum of Akaike weights across all models that include the variable, and it is a useful metric for quantifying the importance of explanatory variables [36]. We present 85% confidence intervals of parameter coefficients, which are fully compatible with information theoretic methods, rather than 95% confidence intervals [37].

Allocation of yolk androgens in Wood Ducks may differ between parasitic and host eggs and potentially influence embryonic developmental rates [38]. Therefore, any differences between temperature treatments in the distribution of parasitic eggs across the laying sequence could influence our results. During daily nest checks, we recorded the laying sequence of eggs and whether 1 egg or > 1 egg had been laid. Nests with > 1 egg indicated they had been parasitized and provided a minimum estimate of brood parasitism (DNA analysis is needed for clear assessment of conspecific brood parasitism). We used a general linear model with binominal response distribution and logit link function (Proc GLIMMIX) to test whether daily parasitism of nests (> 1 egg) across the laying sequence differed between temperature treatments.

We used mixed linear models to examine the relationship between laying sequence and fresh egg mass. We used models with random intercepts and random slopes and grouped eggs within nests. We tested both linear and nonlinear effects of laying sequence on fresh egg mass. Next, we tested the effect of egg laying sequence on body condition of ducklings using mixed linear models. Body mass adjusted for structural size explains more of the variation in total body lipids of day-old Wood Duck ducklings than does body mass alone, so we used residuals from a regression of tarsus (mm) on duckling body mass (g) to estimate body condition of ducklings. Fresh egg mass was used as a covariate in the analysis because of its strong positive effect on duckling mass [30,31]. We wanted to examine whether there was a relationship between body condition of ducklings and laying sequence independent of any differences in egg mass. We included temperature treatment in the models because of its influence on duckling phenotype [30,31]. We used models with random intercepts and random slopes and grouped nests within treatments. Again, we compared models using AIC_c_, ranked models based on their relative differences to the top model (ΔAIC_c)_, and present parameter estimates with 85% confidence intervals. We completed data summaries and analyses using SAS 9.3 [33].

### Ethics statement

The Institutional Animal Care and Use Committee (IACUC) of Auburn University approved our research (PRN 2006–1049). Eggs were collected under permits issued by the U.S. Fish and Wildlife Service (MB 748024–0) and South Carolina (G-10-02).

## Results

**Fig 1:**
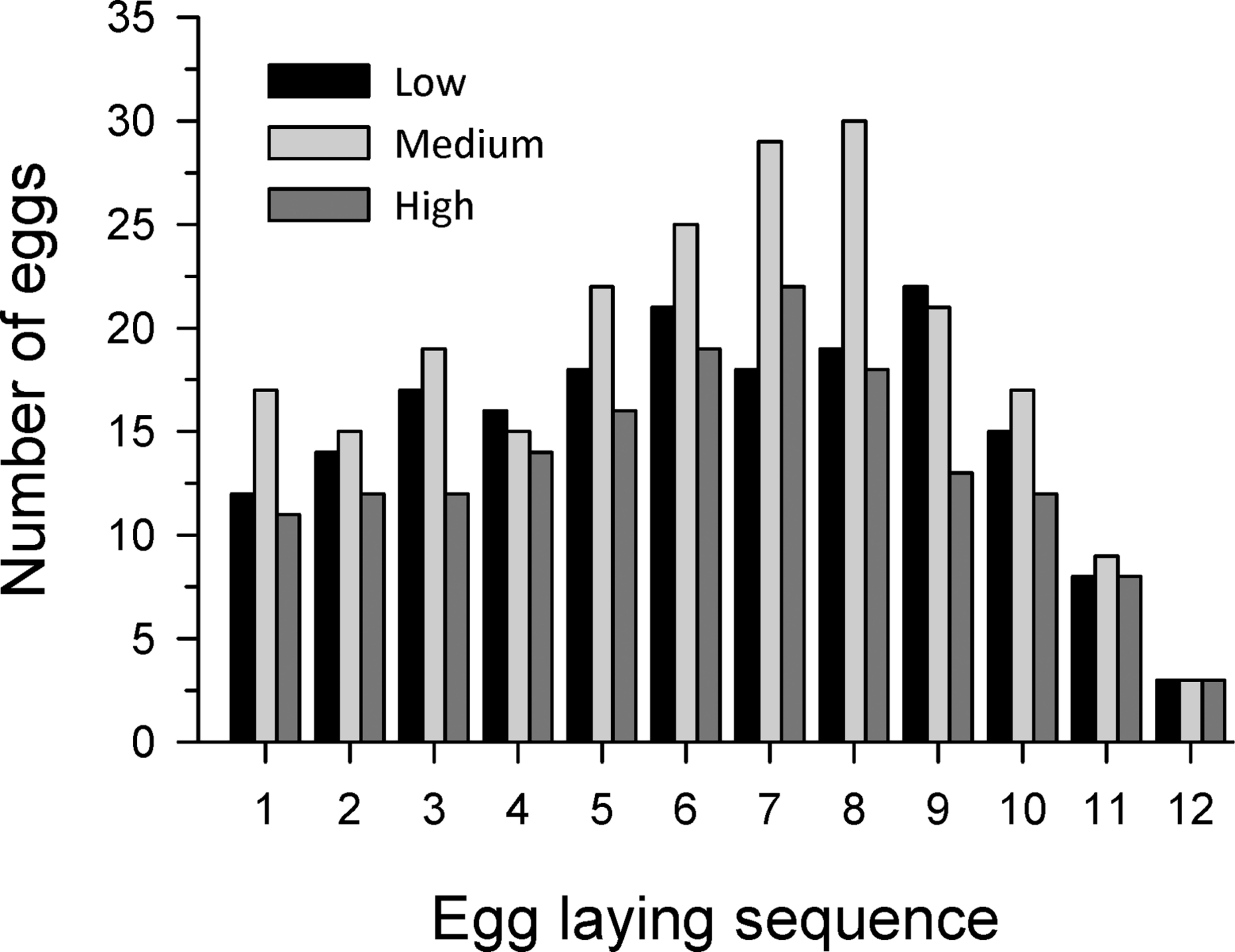
Wood Duck eggs collected and successfully hatched. Frequencies are displayed according to laying sequence (1–12) and incubation temperature (low, medium and high).

**Fig 2:**
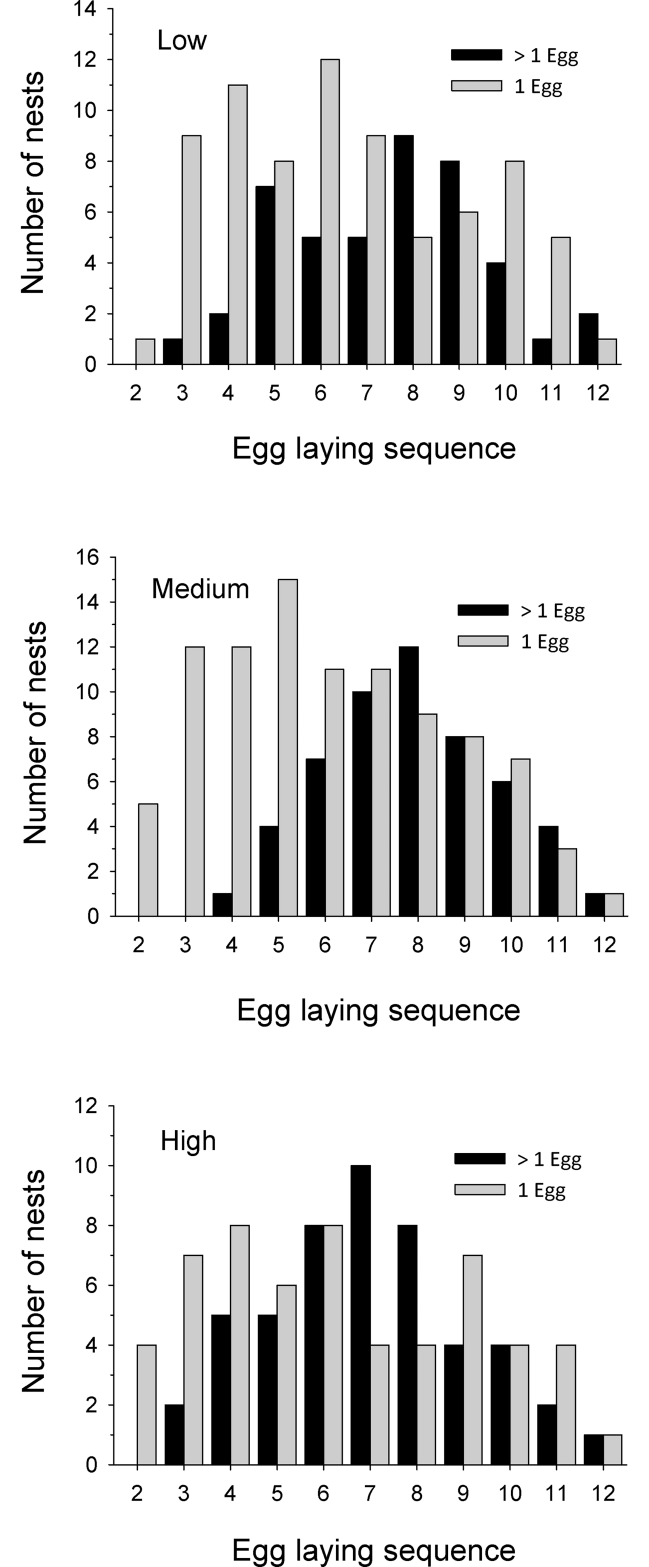
Wood Duck nests containing either 1 or > 1 egg during daily nest checks. Frequencies are displayed by egg laying sequence and incubation temperature (low, medium and high). Only data from active nests that were being checked daily are included.

We collected, incubated, and successfully hatched 565 eggs from 60 nests (Table 1). Nest initiation date averaged 2 April ± 3 d (SE), and nests contained a median of 2 eggs when first discovered, with 60% of nests containing 1 or 2 eggs. Eggs were incubated at three biologically relevant temperatures, and average incubation temperature differed by 2.7°C between high and low treatments (Table 1). Incubation period was about 8 days longer at low incubation temperatures than at high temperatures (Table 1). We used egg-laying sequences of 1 to 12 in our analyses (Fig 1), and only used nests containing eggs with ≥6 laying sequences. Nests were recognized as being parasitized when > 1 egg was laid on at least one day during the laying sequence. Using this criterion, 90% of nests (54 of 60) were parasitized. We visited active nests daily through the laying sequence and recorded days when either 1 or > 1 eggs were found. Number of nests that were parasitized (> 1 egg) on each day of the laying sequence (days 2 to 12) generally occurred less frequently early in the sequence (Fig 2), and the distribution of parasitized and non-parasitized nests across laying sequences did not differ between temperature treatments (*F* = 2.24, df = 2,27, *P* = 0.13).

**Table 1:** Sample sizes of Wood Duck nests and eggs and mean (± SE) incubation temperature and incubation period by temperature treatment.

Treatment	Number of nests	Number of eggs	Incubation temperature, °C	Incubation period, days
Low	21	183	34.9 ± 0.004	37.0 ± 0.09
Medium	23	222	35.8 ± 0.005	33.1 ± 0.09
High	16	160	37.6 ± 0.006	28.7 ± 0.07

### Interactive effect of laying sequence and incubation temperature on incubation period

**Fig 3:**
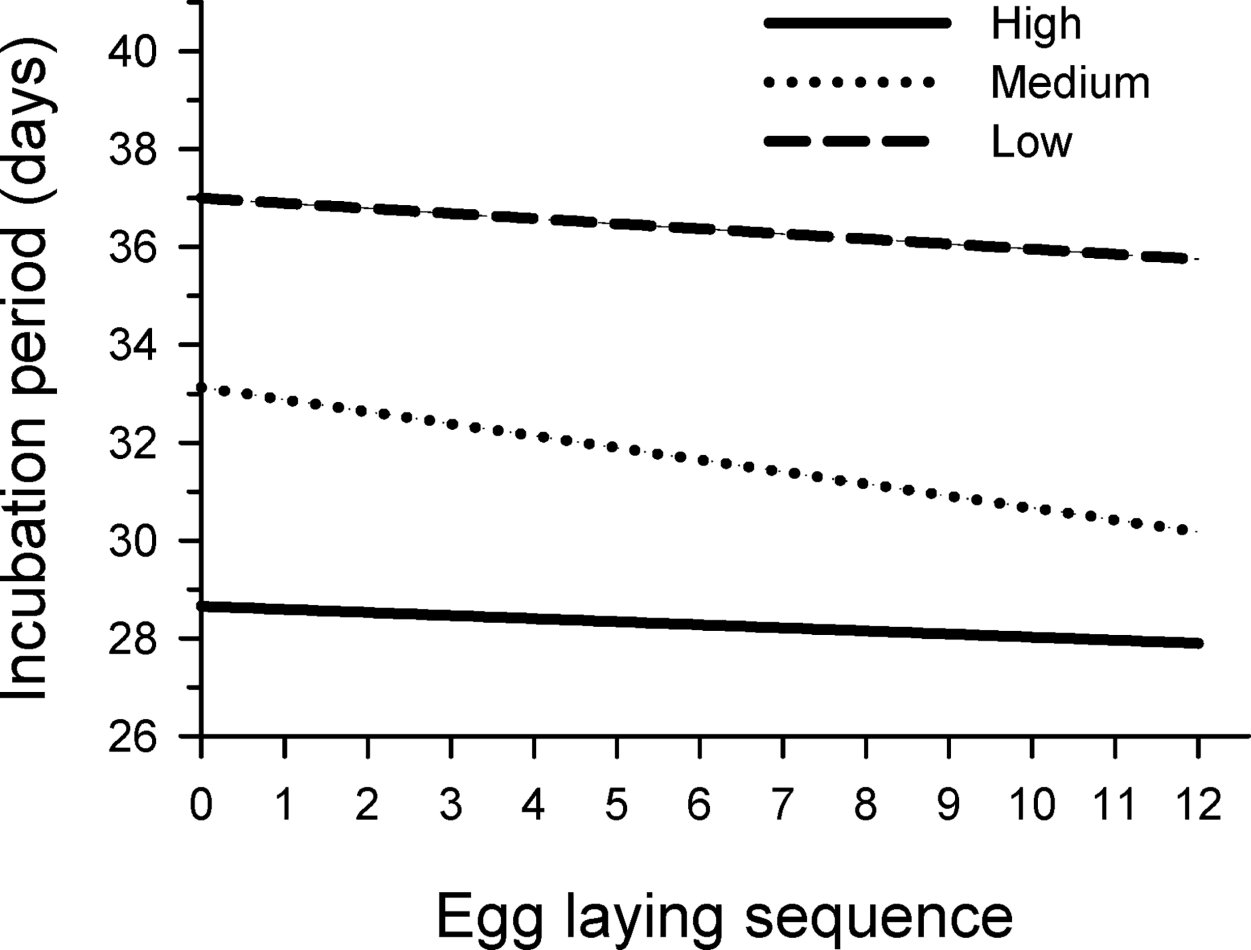
Predicted relationships between egg laying sequence and incubation period. Parameter estimates are from the top ranked model (*w*_*i*_ = 0.76; Table 2) and show interactive effects of egg laying sequence and incubation temperature (low, medium and high) on length of the incubation period in Wood Ducks.

The top ranked model of incubation period (*w*_*i*_ = 0.76) included additive effects of incubation temperature, laying sequence, and the interaction between incubation temperature and laying sequence (Table 2). This model had about 5 times more support than the next best model (*w*_*i*_ = 0.15) which only included incubation temperature and laying sequence, and not their interaction (Table 2). The parameter likelihood values of incubation temperature (1.0), laying sequence (1.0), and fresh egg mass (0.05) showed strong support for including temperature and laying sequence in models of incubation period but little support for including fresh egg mass. Incubation period declined as the laying sequence advanced, but strength of the relationship varied with incubation temperature. Incubation period declined faster as laying sequence progressed for eggs incubated at medium temperatures (ß = -0.25 ± 0.04; 85% CI = -0.30, -0.19) compared to eggs incubated at either low temperatures (ß = -0.10 ± 0.04; 85% CI = -0.16, -0.05) or high temperatures (ß = -0.06 ± 0.04; 85% CI = -0.13, -0.001; Fig 3). The decline in incubation period with laying sequence was slightly greater for eggs incubated at low temperatures than at high temperatures, but 85% CIs of the parameter estimates overlapped. Estimated differences in incubation period between eggs sequenced as 1 and 12 were 2.7 d, 1.2 d, and 0.7 d for eggs incubated at medium, low and high temperatures, respectively.

**Table 2:** Mixed models used to evaluate effect of egg laying sequence on length of the incubation period in Wood Ducks. Incubation temperature and fresh egg mass also were included as potential explanatory variables. We compared models using Akaike’s Information Criterion corrected for small sample size (AIC_c_) and ranked models based on relative differences to the top model (ΔAIC_c_). *w*_*i*_ = Akaike model weight and K = number of parameters.

Rank	Model^a^	ΔAIC_c_	*w*_*i*_	*K*
1	Temperature + Egg sequence + Temperature*Egg sequence	0^b^	0.758	4
2	Temperature + Egg sequence	3.2	0.153	3
3	Temperature + Egg sequence + Egg mass + Temperature*Egg sequence	5.9	0.040	5
4	Temperature + Egg sequence + Egg sequence^2^ + Temperature*Egg sequence	6.2	0.034	5
5	Temperature + Egg sequence + Egg mass	9.0	0.008	4
6	Temperature + Egg sequence + Egg sequence^2^	9.3	0.007	4
7	Temperature	26.0	0.0	2
8	Temperature + Egg mass	31.3	0.0	3
9	Temperature + Egg mass + Temperature*Egg mass	36.8	0.0	4
10	Intercept only	317.8	0.0	1

^a^ Temperature = incubation temperature (low, medium and high); Egg sequence = egg laying sequence (1–12); Egg mass = fresh egg mass (grams).

^b^ AIC_c_ value = 1734.8 for the highest ranking model.

### Influence of laying sequence on egg mass and duckling body condition

The nonlinear model of the relationship between egg mass and laying sequence performed slightly better than the linear model (AIC_c_ = 2871 vs. 2872) and showed that fresh egg mass declined in a nonlinear fashion as the laying sequence advanced (S1 Fig). The best-supported model of duckling condition (*w*_*i*_ = 0.83) included additive effects of incubation temperature and egg mass and had over 6 times more support than the next best model (*w*_*i*_ = 0.13) which included additive effects of incubation temperature, egg mass, and egg sequence (Table 3). Other models of duckling condition had weak support (ΔAIC_c_ ≥ 7.8; Table 3). Parameter likelihood values indicated that incubation temperature (1.0) and fresh egg mass (1.0) had greater relative importance to duckling condition than laying sequence (0.15). The top model showed a strong positive effect of egg mass on duckling condition (ß = 0.50 ± 0.02; 85% CI = 0.47–0.53), and body condition was greatest for ducklings incubated at high temperatures (Least-squares mean: 1.05 ± 0.17) followed by those incubated at medium (-0.15 ± 0.15) and low (-0.79 ± 0.15) temperatures (Table 3, S2 Fig). Model 2 showed a relatively weak decline in duckling body condition as the laying sequence progressed (ß = -0.05 ± 0.02; 85% CI = -0.09, -0.02; Table 3).

**Table 3:** Mixed models used to evaluate effect of egg laying sequence on body condition of newly hatched Wood Ducks. Incubation temperature and fresh egg mass also were included as potential explanatory variables. We compared models using Akaike’s Information Criterion corrected for small sample size (AIC_c_) and ranked models based on relative differences to the top model (ΔAIC_c_). *w*_*i*_ = Akaike model weight and *K* = number of parameters.

Rank	Model^a^	ΔAIC_c_	*w*_*i*_	*K*
1	Temperature + Egg mass	0^b^	0.833	3
2	Temperature + Egg mass + Egg sequence	3.7	0.131	4
3	Temperature + Egg mass + Temperature*Egg mass	7.8	0.017	4
4	Temperature + Egg mass + Egg sequence + Egg sequence^2^	9.7	0.007	5
5	Temperature + Egg mass + Egg mass^2^	10.0	0.006	4
6	Temperature + Egg mass + Egg sequence + Temp*Egg sequence	10.7	0.004	5
7	Temperature + Egg mass + Egg sequence + Temp*Egg mass	11.5	0.003	5
8	Temperature + Egg mass + Egg sequence + Egg mass^2^	13.6	0.001	5
9	Egg mass	41.7	0.0	2
10	Temperature + Egg sequence	393.9	0.0	3
11	Temperature + Egg sequence + Temperature*Egg sequence	399.4	0.0	4
12	Temperature	400.5	0.0	2
13	Temperature + Egg sequence + Egg sequence^2^	401.0	0.0	4
14	Egg sequence	419.3	0.0	2
15	Intercept only	430	0.0	1

^a^ Temperature = incubation temperature (low, medium and high); Egg mass = fresh egg mass (grams); Egg sequence = egg laying sequence (1–12)

^b^ AIC_c_ value = 2087.3 for the highest ranking model.

## Discussion

Incubation period of Wood Ducks declined with increasing incubation temperature and was shortest for eggs incubated at high temperatures, which is similar to naturally-incubated clutches and is consistent with our previous studies [30,31]. Incubation period also declined as the egg laying sequence progressed, but strength of the relationship was affected by incubation temperature. Accelerated development of eggs as the laying sequence progressed was stronger for eggs incubated at intermediate temperatures (35.8°C) than for eggs incubated at high (37.6°C) and low (34.9°C) temperatures. At intermediate incubation temperatures, estimated differences in development time between the first and last egg in a 12-egg laying sequence closely matched the amount of developmental asynchrony that occurs in naturally incubated Wood Duck clutches as full incubation begins [17,29]. We suggest differences in rate of embryo development between early- and late-laid eggs were sufficient to eliminate developmental asynchrony within clutches only for eggs incubated at intermediate temperatures and not for those incubated at high or low temperatures. Body condition of ducklings declined slightly with laying sequence and may represent a potential cost of faster development; however, both incubation temperature and fresh egg mass had much stronger effects on duckling condition than did laying sequence.

### Importance of incubation temperature on avian development

The temperature that avian parents incubate eggs can impact reproductive success by influencing neonate phenotypes and predation risk [3]. In Wood Ducks, for example, low incubation temperature negatively influences a suite of neonate characteristics like immune function, body condition, locomotor ability, and thermoregulation that can affect survival and reproductive success [3,4,31]. Harmful effects of low incubation temperature on neonate phenotypes are not unique to Wood Ducks, but have also been reported in a variety of avian species including Australian Brush-turkeys (*Alectura lathami*) [39], Zebra Finches (*Taeniopygia guttata*) [2,40], Tree Swallows (*Tachycineta bicolor*) [41], and Blue Tits (*Cyanistes caeruleus*) [42].

High incubation temperatures also negatively affect young birds, but consequences may not occur immediately [7]. Adverse effects of high incubation temperature are related to high embryonic growth rates that result in increased oxidative damage and produce low quality adults with reduced survival [5,6]. Accelerated growth of nestlings, for example, resulted in increased oxidative damage in Zebra Finches [43] and Coal Tits (*Periparus ater*) [44], and increased oxidative damage in nestling European Shags (*Phalacrocorax aristotelis*) was linked to reduced post-fledging survival [45]. In Wood Ducks, embryos incubated at higher temperatures developed faster and had greater daily metabolic rates than embryos incubated at low temperatures [46]. However, young Wood Ducks from eggs incubated at higher temperatures had greater survival and reproductive success than those incubated at low temperatures [31]. Therefore, it is clear the relationships between incubation temperature, offspring quality, and fitness in birds are complex and deserve further study [4].

### Embryo development, laying sequence and temperature

We found incubation period of Wood Ducks declined linearly as laying sequence progressed which is also true in Black Brant (*Branta bernicla nigricans*) [20]. Mechanisms responsible for faster development of late-laid eggs are not entirely clear but may include intrinsic factors like increased levels of yolk androgens (e.g., testosterone (T), androstenedione (A_4_) and 5α-dihydrotestosterone (5α-DHT); [23,24,47,48]), changes in egg size and composition [26,49], and increased embryonic metabolic rates [21]. However, we found differences in egg mass had little effect on development rate in Wood Ducks. New integrative studies (e.g., [50]) are needed to clarify the role that these or possibly other mechanisms have in accelerating development of late-laid eggs in precocial species.

We could not clearly differentiate parasitic eggs from those of host females, so we used eggs of both parasites and hosts in our analyses. Doing so may have influenced our results in two important ways. First, it produced greater uncertainty in assigning laying order to eggs within nests, which potentially weakened the relationship between laying sequence and incubation period. Second, intrinsic quality of eggs is known to influence rate of embryo development and can vary with laying sequence and between host and parasitic females [23–26]. In Wood Ducks, for example, Odell [38] found that androstenedione (A_4_) increased as the laying sequence advanced in eggs of hosts but not of parasites and speculated that increased levels of A_4_ facilitated hatching synchrony. Potential differences in egg quality, therefore, make it is especially important that laying sequence of brood parasites match those of hosts to facilitate synchronous embryo development and hatching in precocial birds. In our study, if egg sequences of hosts and parasites were mismatched, this too would have weakened our ability to detect laying sequence effects on incubation period. Ultimately, molecular genetic data will be needed to separate eggs of parasites from those of hosts to confirm our results. Nevertheless, we believe the pattern of declining incubation period as the laying sequence progressed in Wood Ducks was real. Indeed, these results suggest that parasitic females were able to closely match egg quality and laying sequences of their hosts. In one of the few waterfowl studies, Lemons and Sedinger [51] also reported that Black Brant brood parasites were able to recognize and closely match egg size of their hosts. Matching of the host’s laying sequence by parasites has been reported in several non-waterfowl species, but more investigations of this phenomenon are especially needed in precocial birds [52].

In our study, incubation temperature interacted with laying order which was not surprising given the strong effect incubation temperature has on embryo development [3,4]. Only intermediate incubation temperatures provided the necessary thermal environment that allowed late-laid eggs to adequately accelerate development, reduce developmental asynchrony, and potentially synchronize hatching with early-laid eggs. At both high and low incubation temperatures, differences in developmental rates between the first and last laid egg would not completely compensate for natural levels of developmental asynchrony in Wood Duck clutches, which averages 2.2 d [17].

Why should temperature matter? Incubating parents attempt to keep eggs at optimal temperatures, but trade-offs between the needs of parents and offspring are important in helping to shape this relationship. We know that incubation temperature has a strong effect on rate of embryo development in a variety of birds including Wood Ducks [30,53]. In this study, incubation periods at high temperatures (29 d) were four days shorter than at intermediate temperatures (33 d). In naturally incubated Wood Duck nests, however, incubation periods as short as 28–29 d are typically rare [30,32,54,55]. As we have noted, faster embryonic development can be costly by increasing oxidative stress and reducing offspring quality [5]. Additional increases in developmental rates of late-laid eggs at high incubation temperatures may impose costs to neonates that cannot be balanced by potential fitness gains associated with reductions in asynchrony. There also may be intrinsic constraints to further increasing development rates of late-laid eggs at high incubation temperatures. For example, metabolic rates of embryos increase as developmental rates increase and can be greater both for eggs laid later in the sequence (Canada Goose; [21]) and for eggs incubated at higher temperatures (Wood Duck; [46]). Because rates of embryonic growth and metabolism of late-laid eggs are already high, the potential for further increases at high incubation temperatures may be limited. In songbirds that naturally incubate eggs at high temperatures, for example, rates of embryonic development increased only slightly when nests were experimentally warmed [56]. It is likely that intrinsic constraints and costs to neonates of faster growth have been important in establishing an upper limit to rate of embryonic development.

As for low incubation temperature, we know low temperatures negatively affect embryonic development and a variety of phenotypic traits of Wood Duck neonates that carry over and reduce post-hatch survival and future reproduction [3,4]. Therefore, it was not surprising that accelerated development of late-laid eggs incubated at low temperatures did not completely compensate for normal levels of within-clutch developmental asynchrony. This outcome is another example of how low incubation temperature represents a suboptimal environment for embryo development.

### Maintaining optimal incubation temperatures

Optimum incubation temperatures occur within a narrow range for most birds [1]. Small variations above and below the optimum temperature can affect reproductive success of birds by influencing rates of embryo development and neonate phenotypes [3,4,7]. Parents influence the thermal environment of eggs through changes in the size, structure and composition of nests [57–59], nest site location and orientation [60,61], timing of nesting [62–64], and incubation behavior [32]. There also is a positive relationship between ambient temperature and incubation temperature [32,64]. As ambient temperatures increase, incubating parents frequently spend less time on the nest and experience reduced incubation costs while maintaining optimum incubation temperatures [32]. However, high ambient temperatures may be costly if incubation temperatures increase too much. In our study, late-laid eggs incubated at the highest temperature (37.6°C) did not develop fast enough to fully mitigate natural levels of within-clutch developmental asynchrony. Furthermore, developmental asynchrony within clutches may actually increase with increasing ambient temperatures. In Zebra Finches, for example, high ambient temperatures caused increased levels of ‘ambient incubation’ of early-laid eggs that produced greater levels of hatching asynchrony [65]. In King Rails (*Rallus elegans*), as the breeding season advanced and ambient temperatures increased, parents began incubation earlier during egg laying which resulted in increased hatching asynchrony [64]. Future research should explore the potential importance of predicted changes to climate on developmental asynchrony and reproductive success in precocial birds.

## Supporting information

S1 Fig Egg laying sequence and fresh egg mass. Nonlinear relationship (± 85% CI) between egg laying sequence and fresh egg mass of Wood Ducks at the Savannah River Site, South Carolina.
(TIFF)

S2 Fig Incubation temperature and duckling body condition. We present body condition (Least-squares mean ± SE) of newly hatched Wood Ducks as the residuals from a regression of tarsus length (mm) on body mass (g). These residuals are better predictors of duckling lipids than body mass alone.
(TIFF)

## References

[1] Webb DR. Thermal tolerance of avian embryos: a review. The Condor 1987; 89:874–898.

[2] Olson CR, Vleck CM, Vleck D. Periodic cooling of bird eggs reduces embryonic growth efficiency. Physiological and Biochemical Zoology 2006; 79:927–936. doi: 10.1086/506003 16927239 10.1086/506003

[3] DuRant SE, Hopkins WA, Hepp GR, Walters JR. Ecological, evolutionary, and conservation implications of incubation temperature‐dependent phenotypes in birds. Biological Reviews 2013; 88:499–509. doi: 10.1111/brv.12015 23368773 10.1111/brv.12015

[4] Hepp GR, DuRant SE, Hopkins WA. Influence of incubation temperature on offspring phenotype and fitness in birds In: Deeming DC, Reynolds SJ, editors. Nests, eggs, and incubation: new ideas about avian reproduction. Oxford University Press; 2015 pp. 171–178.

[5] Ricklefs RE. Embryo development and ageing in birds and mammals. Proceedings of the Royal Society B 2006; 273:2077–2082. doi: 10.1098/rspb.2006.3544 16846916 10.1098/rspb.2006.3544 PMC1635478

[6] Monaghan P, Metcalfe NB, Torres R. Oxidative stress as a mediator of life history trade-offs: mechanisms, measurements and interpretation. Ecology Letters 2009; 12:75–92. doi: 10.1111/j.1461-0248.2008.01258.x 19016828 10.1111/j.1461-0248.2008.01258.x

[7] Nord A, Nilsson J-Å. Long-term consequences of high incubation temperature in a wild bird population. Biology Letters 2016; 12:20160087 doi: 10.1098/rsbl.2016.0087 27048468 10.1098/rsbl.2016.0087 PMC4881354

[8] Wang JM, Beissinger SR. Variation in the onset of incubation and its influence on avian hatching success and asynchrony. Animal Behaviour 2009; 78:601–613.

[9] Wang JM, Beissinger SR. Partial incubation in birds: its occurrence, function, and quantification. The Auk 2011; 128:454–466.

[10] Loos ER, Rohwer F. Laying-stage nest attendance and onset of incubation in prairie nesting ducks. The Auk 2004; 121:587–599.

[11] Arnold TW, Rohwer FC, Armstrong T. Egg viability, nest predation, and the adaptive significance of clutch size in prairie ducks. American Naturalist 1987; 130:643–653.

[12] Stoleson SH, Beissinger SR. Egg viability as a constraint on hatching synchrony at high ambient temperatures. Journal of Animal Ecology 1999; 68:951–962.

[13] Beissinger SR, Cook MI, Arendt W. The shelf life of bird eggs: testing egg viability using a tropical climate gradient. Ecology 2005; 86:2164–2175.

[14] Walls JG, Hepp GR, Eckhardt LG. Effects of incubation delay on viability and microbial growth of Wood Duck (*Aix sponsa*) eggs. The Auk 2011; 128:663–670.

[15] Lack D. Population studies of birds Clarendon Press; 1966.

[16] Stoleson SH, Beissinger SR. Hatching asynchrony, brood reduction, and food limitation in a neotropical parrot. Ecological Monographs 1997; 67:131–154.

[17] Kennamer RA, Harvey WF, Hepp GR. Embryonic development and nest attentiveness of Wood Ducks during egg laying. The Condor 1990; 92:587–592.

[18] Flint PL, Lindberg MS, MacCluskie MC, Sedinger JS. The adaptive significance of hatching synchrony in waterfowl eggs. Wildfowl 1994; 45:248–254.

[19] Vince MA. Social facilitation of hatching in the Bobwhite Quail. Animal Behaviour 1964; 12:531–534.

[20] Nicolai CA, Sedinger JS, Wege ML. Regulation of development time and hatch synchronization in Black Brant (*Branta bernicla nigricans*). Functional Ecology 2004; 18:475–482.

[21] Boonstra TA, Clark ME, Reed WL. Position in the sequence of laying, embryonic metabolic rate, and consequences for hatching synchrony and offspring survival in Canada Geese. The Condor 2010; 112:304–313.

[22] Davies JC, Cooke F. Intraclutch hatch synchronization in the Lesser Snow Goose. Canadian Journal of Zoology 1983; 61:1398–1401.

[23] Schwabl H. Yolk is a source of maternal testosterone for developing birds. Proceedings of the National Academy of Sciences of the USA 1993; 90:11439–114411. 8265571 10.1073/pnas.90.24.11446 PMC48000

[24] Schwabl H, Palacios MG, Martin TE. Selection for rapid embryo development correlates withembryo exposure to maternal androgens among passerine birds. American Naturalist 2007; 170:196–206. doi: 10.1086/519397 17874371 10.1086/519397

[25] Flint PL, Sedinger JS. Reproductive implications of egg size variation in the Black Brant. The Auk 1992; 109:896–903.

[26] Kennamer RA, Alsum SK, Colwell SV. Composition of Wood Duck eggs in relation to egg size, laying sequence, and skipped days of laying. The Auk 1997; 114:479–487.

[27] Groothuis TGG, Eising CM, Dijkstra C, Müller W. Balancing between costs and benefits of maternal hormone deposition in avian eggs. Biology Letters 2005; 1:78–81. doi: 10.1098/rsbl.2004.0233 17148133 10.1098/rsbl.2004.0233 PMC1629043

[28] Hepp GR, Bellrose FC. Wood Duck (*Aix sponsa*) In: Rodewald PG, editor. The Birds of North America. Cornell Lab of Ornithology; 2013 doi: 10.2173/bna.169

[29] Hepp GR. Early onset of incubation in Wood Ducks. The Condor 2004; 106:182–186.

[30] Hepp GR, Kennamer RA, Johnson MH. Maternal effects in Wood Ducks: incubation temperature influences incubation period and neonate phenotype. Functional Ecology 2006; 20:307–314.

[31] Hepp GR, Kennamer RA. Warm is better: incubation temperature influences apparent survival and recruitment of Wood Ducks (*Aix sponsa*). PLoS ONE 2012; 7:e47777 doi: 10.1371/journal.pone.0047777 23077669 10.1371/journal.pone.0047777 PMC3471843

[32] McClintock ME, Hepp GR, Kennamer RA. Plasticity of incubation behaviors helps Wood Ducks (*Aix sponsa*) maintain an optimal thermal environment for developing embryos. The Auk: Ornithological Advances 2014; 131: 672–680.

[33] SAS Institute. SAS/STAT 9.3 User’s Guide. SAS Institute; 2011.

[34] Schielzeth H, Forstmeier W. Conclusions beyond support: overconfident estimates in mixed models. Behavioral Ecology 2009; 20:416–420. doi: 10.1093/beheco/arn145 19461866 10.1093/beheco/arn145 PMC2657178

[35] Burnham KP, Anderson DR. Model selection and multinominal inference–a practical information theoretic approach, 2^nd^ ed. New York: Springer-Verlag; 2002.

[36] Giam X, Olden JD. Quantifying variable importance in a multimodel inference framework. Methods in Ecology and Evolution 2016; 7:388–397.

[37] Arnold TW. Uniformative parameters and model selection using Akaike’s information criterion. Journal of Wildlife Management 2010; 74:1175–1178.

[38] Odell NS. Female reproductive investment in a conspecific brood parasite. Ph.D. Dissertation, University of California, Davis; 2008.

[39] Eiby YA, Booth DT. The effects of incubation temperature on the morphology and composition of Australian Brush-turkey (*Alectura lathami*) chicks. Journal of Comparative Physiology B 2009; 179:875–882. 10.1007/s00360-009-0370-4 19471897

[40] Berntsen HH, Bech C. Incubation temperature influences survival in a small passerine bird. Journal of Avian Biology 2016; 46:141–145.

[41] Ardia DR, Pérez JH, Clotfelter ED. Experimental cooling during incubation leads to reduced innate immunity and body condition of nestling Tree Swallows. Proceedings of the Royal Society B: Biological Sciences 2010; 277:1881–1888. doi: 10.1098/rspb.2009.2138 20147326 10.1098/rspb.2009.2138 PMC2871872

[42] Nord A, Nilsson J-Å. Incubation temperature affects growth and energy metabolism in Blue Tit nestlings. The American Naturalist 2011; 178:639–651. doi: 10.1086/662172 22030733 10.1086/662172

[43] Alonso-Alvarez C, Bertrand S, Faivre B, Sorci G. Increased susceptibility to oxidative damage as a cost of accelerated growth in Zebra Finches. Functional Ecology 2007; 21:873–879.

[44] Stier A, Delestrade A, Zahn S, Arrivé M, Criscuolo F, Massemin-Challet S. Elevation impacts the balance between growth and oxidative stress in Coal Tits. Oecologia 2014; 175:791–800. doi: 10.1007/s00442-014-2946-2 24805201 10.1007/s00442-014-2946-2

[45] Noguera JC, Kim S-Y, Velando A. Pre-fledgling oxidative damage predicts recruitment in a long-lived bird. Biology Letters 2012; 8:61–63. doi: 10.1098/rsbl.2011.0756 21865247 10.1098/rsbl.2011.0756 PMC3259982

[46] DuRant SE, Hopkins WA, Hepp GR. Embryonic developmental patterns and energy expenditure are affected by incubation temperature in Wood Ducks (*Aix sponsa*). Physiological and Biochemical Zoology 2011; 84:451–457. doi: 10.1086/661749 21897082 10.1086/661749

[47] Eising CM, Eikenaar C, Schwabl H, Groothuis TGG. Maternal androgens in black-headed gull (*Larus ridibundus*) eggs: consequences for chick development. Proceedings of the Royal Society B: Biological Sciences 2001; 268:839–846. doi: 10.1098/rspb.2001.1594 11345330 10.1098/rspb.2001.1594 PMC1088678

[48] Gorman KB, Williams TD. Correlated evolution of maternally derived yolk testosterone and early developmental traits in passerine birds. Biology Letters 2005; 1:461–464. doi: 10.1098/rsbl.2005.0346 17148233 10.1098/rsbl.2005.0346 PMC1626382

[49] Arnold TW. Factors affecting egg viability and incubation time in prairie dabbling ducks. Canadian Journal of Zoology 1993; 71:1146–1152.

[50] Williams TD, Groothuis TGG. Egg quality, embryonic development, and post-hatching phenotype: an integrated perspective In: Deeming DC, Reynolds SJ, editors. Nests, eggs, and incubation: new ideas about avian reproduction. Oxford University Press; 2015 pp. 113–126.

[51] Lemons PR, Sedinger JS. Egg size matching by an intraspecific brood parasite. Behavioral Ecology 2011; 22:696–700.

[52] Pӧysӓ H, Eadie, JM, Lyon, BE. Conspecific brood parasitism in waterfowl and cues parasites use. In: Rees, E, Kaminski, R, Webb, L, editors. Ecology and conservation of waterfowl in the Northern Hemisphere. Wildfowl Special Issue no. 4; 2014. pp. 192–21.

[53] Martin TE, Ton R, Niklison A. Intrinsic vs. extrinsic influences on life history expression: metabolism and parentally induced temperature influences on embryo development rate. Ecology Letters 2013; 16:738–745. doi: 10.1111/ele.12103 23473270 10.1111/ele.12103

[54] Manlove CA, Hepp GR. Patterns of nest attendance in female Wood Ducks. The Condor 2000; 102:286–291.

[55] Folk TH, Hepp GR. Effects of habitat use and movement patterns on incubation behavior of female Wood Ducks (*Aix sponsa*) in southeast Alabama. The Auk 2003; 120:1159–1167.

[56] Ton R, Martin TE. Proximate effects of temperature versus evolved intrinsic constraints for embryonic development times among temperate and tropical songbirds. Scientific Reports 2017; 7: 895 doi: 10.1038/s41598-017-00885-3 28420877 10.1038/s41598-017-00885-3 PMC5429855

[57] Rohwer VG, Law JSY. Geographic variation in nests of yellow warblers breeding in Churchill, Manitoba, and Elgin, Ontario. The Condor 2010; 112:596–604.

[58] Hilton GM, Hansell MH, Ruxton GD, Reid JM, Monaghan P. Using artificial nests to test importance of nesting material and nest shelter for incubation energetics. The Auk 2004; 121:777–787.

[59] Windsor RL, Fegely JL, Ardia DR. The effects of nest size and insulation on thermal properties of Tree Swallow nests. Journal of Avian Biology 2013; 44:305–310.

[60] Nelson KJ, Martin K. Thermal aspects of nest-site location for Vesper Sparrows and Horned Larks in British Columbia. Studies in Avian Biology 1999; 19:137–143.

[61] Ardia DR, Pérez JH, Clotfelter ED. Nest box orientation affects internal temperature and nest site selection by Tree Swallows. Journal of Field Ornithology 2006; 77:339–344.

[62] Hepp GR, Kennamer RA. Date of nest initiation mediates incubation costs of Wood Ducks (*Aix sponsa*). The Auk 2011; 128:254–264.

[63] Coe BH, Beck ML, Chin SY, Jachowski CMB, Hopkins WA. Local variation in weather conditions influences incubation behavior and temperature in a passerine bird. Journal of Avian Biology 2015; 46:385–394.

[64] Clauser AJ, McRae SB. Plasticity in incubation behavior and shading by King Rails *Rallus elegans* in response to temperature. Journal of Avian Biology 2017; 48:479–488.

[65] Griffith SC, Mainwaring MC, Sorato E, Beckmann C. High atmospheric temperatures and ‘ambient incubation’ drive embryonic development and lead to earlier hatching in a passerine bird. Royal Society Open Science 2016; 3:150371 doi: 10.1098/rsos.150371 26998315 10.1098/rsos.150371 PMC4785966

