# Supplementary figures and images for: Laying sequence interacts with incubation temperature to influence rate of embryonic development and hatching synchrony in a precocial bird
Source: PLoS One. 2018 Jan 26;13(1):e0191832. doi: 10.1371/journal.pone.0191832 (PMC5786303; doi:10.1371/journal.pone.0191832)

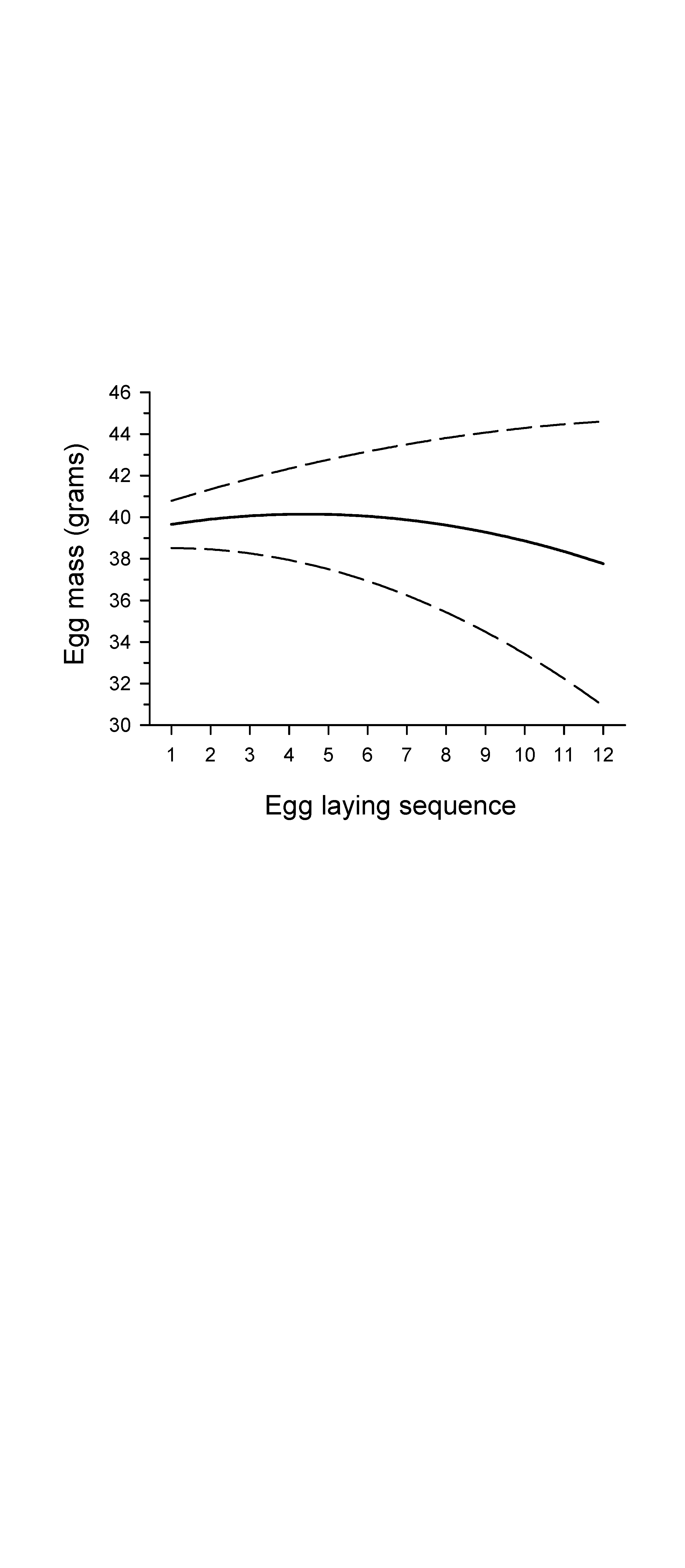

Supplement: S1 Fig — Nonlinear relationship (± 85% CI) between egg laying sequence and fresh egg mass of Wood Ducks at the Savannah River Site, South Carolina. (TIFF) [file pone.0191832.s001.tiff]

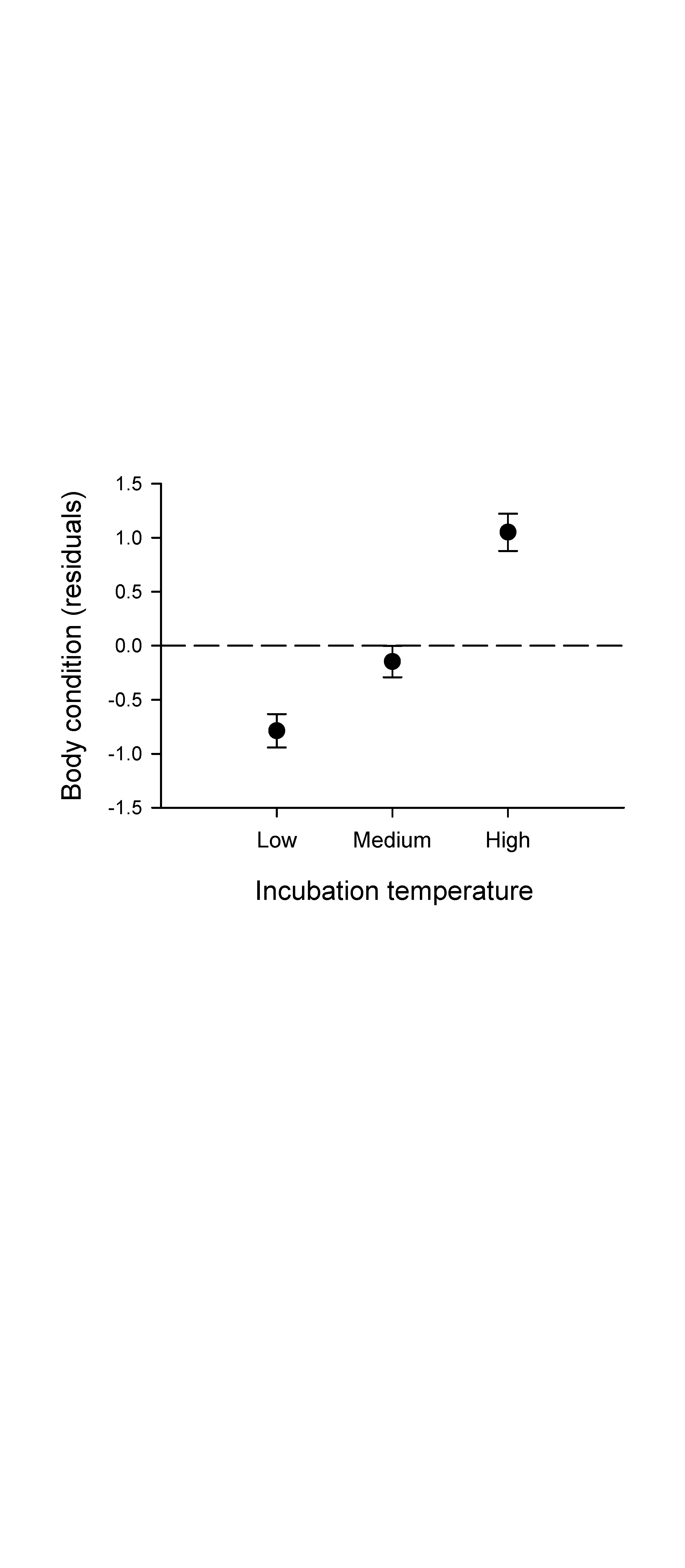

Supplement: S2 Fig — We present body condition (Least-squares mean ± SE) of newly hatched Wood Ducks as the residuals from a regression of tarsus length (mm) on body mass (g). These residuals are better predictors of duckling lipids than body mass alone. (TIFF) [file pone.0191832.s002.tiff]
